# NTRK Gene Fusion Detection in Atypical Spitz Tumors

**DOI:** 10.3390/ijms222212332

**Published:** 2021-11-15

**Authors:** Rocco Cappellesso, Filippo Nozzoli, Federica Zito Marino, Sara Simi, Francesca Castiglione, Vincenzo De Giorgi, Carlo Cota, Rebecca Senetta, Giosuè Scognamiglio, Anna Maria Anniciello, Anna Maria Cesinaro, Mario Mandalà, Andrea Gianatti, Maria Gabriella Valente, Barbara Valeri, Angela Rita Sementa, Costantino Ricci, Barbara Corti, Giandomenico Roviello, Angelo Paolo Dei Tos, Renato Franco, Daniela Massi

**Affiliations:** 1 Pathological Anatomy Unit, University Hospital of Padua, 35121 Padua, Italy; rocco.cappellesso@gmail.com (R.C.); angelo.deitos@unipd.it (A.P.D.T.); 2 Section of Anatomic Pathology, Department of Health Sciences, University of Florence, 50139 Florence, Italy; filippo.nozzoli@unifi.it (F.N.); sara.simi@unifi.it (S.S.); 3 Pathology Unit, University of Campania “L. Vanvitelli”, 80129 Naples, Italy; federica.zitomarino@unicampania.it (F.Z.M.); renato.franco@unicampania.it (R.F.); 4 Section of Anatomic Pathology, Careggi University Hospital, 50139 Florence, Italy; francesca.castiglione@gmail.com; 5 Department of Dermatology, University of Florence, 50139 Florence, Italy; vincenzo.degiorgi@unifi.it; 6 Unit of Dermatopathology, San Gallicano Dermatological Institute, 00153 Rome, Italy; carlo.cota@info.gov.it; 7 Pathology Unit, Department of Oncology, University of Turin, 10126 Turin, Italy; rebesenetta@gmail.com; 8 Pathology Unit, Istituto Nazionale Tumori-IRCCS -Fondazione G Pascale, 80131 Naples, Italy; giosco80@gmail.com (G.S.); a.anniciello@istitutotumori.na.it (A.M.A.); 9 Department of Anatomic Pathology, Azienda Ospedaliero-Universitaria di Modena, 41122 Modena, Italy; cesinaro.annamaria@policlinico.mo.it; 10 Unit of Medical Oncology, University of Perugia, 06129 Perugia, Italy; mario.mandala@unipg.it; 11 Pathology Unit, ASST-Papa Giovanni XXIII, 24127 Bergamo, Italy; agianatti@asst-pg23.it; 12 Department of Pathology, San Gerardo Hospital, University of Milano-Bicocca, 20900 Monza, Italy; mg.valente@asst-monza.it; 13 Department of Pathology, Fondazione IRCCS Istituto Nazionale dei Tumori di Milano, 20133 Milan, Italy; Barbara.Valeri@istitutotumori.mi.it; 14 Pathology Unit, IRCCS Istituto Giannina Gaslini, 16147 Genoa, Italy; angelaritasementa@gaslini.org; 15 Pathology Unit, Ospedale Maggiore, 40133 Bologna, Italy; costanricci@gmail.com; 16 Department of Experimental, Diagnostic and Specialty Medicine (DIMES), University of Bologna, 40126 Bologna, Italy; 17 Pathology Unit, IRCCS University Hospital of Bologna, 40133 Bologna, Italy; barbara.corti@aosp.bo.it; 18 Department of Health Sciences, University of Florence, 50139 Florence, Italy; giandomenico.roviello@unifi.it; 19 Surgical Pathology and Cytopathology Unit, Department of Medicine (DIMED), University of Padua, 35128 Padua, Italy

**Keywords:** atypical spitz tumor, *NTRK*, *NTRK1*, *NTRK3*, pan-TRK

## Abstract

Atypical Spitz tumors (AST) deviate from stereotypical Spitz nevi for one or more atypical features and are now regarded as an intermediate category of melanocytic tumors with uncertain malignant potential. Activating *NTRK1/NTRK3* fusions elicit oncogenic events in Spitz lesions and are targetable with kinase inhibitors. However, their prevalence among ASTs and the optimal approach for their detection is yet to be determined. A series of 180 ASTs were screened with pan-TRK immunohistochemistry and the presence of *NTRK* fusions was confirmed using FISH, two different RNA-based NGS panels for solid tumors, and a specific real time RT-PCR panel. Overall, 26 ASTs showed pan-TRK immunostaining. *NTRK1* fusions were detected in 15 of these cases showing cytoplasmic immunoreaction, whereas *NTRK3* was detected in one case showing nuclear immunoreaction. Molecular tests resulted all positive in only two ASTs (included the *NTRK3* translocated), RNA-based NGS and real time RT-PCR were both positive in three cases, and FISH and real time RT-PCR in another two cases. In seven ASTs *NTRK1* fusions were detected only by FISH and in two cases only by real time RT-PCR. The frequency of NTRK fusions in ASTs is 9%, with a clear prevalence of *NTRK1* compared to *NTRK3* alterations. Pan-TRK immunohistochemistry is an excellent screening test. Confirmation of *NTRK* fusions may require the use of different molecular techniques.

## 1. Introduction

The term atypical Spitz tumor (AST) identifies a heterogeneous group of melanocytic lesions with clinical, histopathological, and molecular features overlapping those of benign Spitz nevus and Spitz melanoma [1]. The real incidence of ASTs is currently unknown, probably because of the lack of standardized terminology and the blurred interpretative criteria used in the past. Nevertheless, ASTs have been reported to account approximately 6–8% of the overall number of Spitz nevi [1]. ASTs can occur at any age, but they are more common among adolescents and adults younger than 40 years of age [2] ASTs usually present on the extremities and the trunk as amelanotic or hypopigmented and rarely ulcerated cutaneous plaques or nodules of 5 to 10 mm [2]. Histologically, ASTs are regarded as an intermediate category of melanocytic lesions composed of epithelioid or spindle melanocytes with ground-glass cytoplasm, showing some additional worrisome features such as increased size, asymmetry, ulceration, partial or complete lack of maturation, solid growth, greater pagetoid spread and deeper extension than in Spitz nevus, cytological pleomorphism, increased mitotic activity, and deep and atypical mitoses [2,3,4]. The high inter-observer discrepancy in AST diagnosis, even among internationally recognized dermatopathology experts, is due to the possible different interpretations of these variables [3,4].

The molecular landscape of spitzoid neoplasms is peculiar. Unlike most common and congenital nevi and malignant melanomas, they lack oncogenic *BRAF* and *NRAS* mutations and harbor tumorigenic *HRAS* mutations or kinase gene fusions involving *ALK, BRAF, MET, NTRK1, NTRK3, RET,* and *ROS1* in a mutually exclusive pattern [5,6,7,8,9,10,11,12,13,14,15,16,17]. Due to the different inclusion criteria and diverse methods in the literature, it is difficult to estimate the frequency of *NTRK* fusions in ASTs. Wiesner and colleagues first reported the occurrence of fusions involving one of the *NTRK* genes in ASTs [13]. They found that eight out of thirty-two (25%) ASTs harbored *NTRK1* fusions, as demonstrated by RNA-based NGS, qRT-PCR, and FISH [13]. The identified fusions were *LMNA-NTRK1* and *TP53-NTRK1*, the former being the most common [13]. For comparison, *NTRK* fusions were detected in eight out of seventy-five (11%) Spitz nevi and in seven out of thirty-three (25%) spitzoid melanomas [18]. Amin and colleagues studied 43 ASTs with FISH-confirmed gene fusions involving *ALK, NTRK1, BRAF,* or *RET* [17]. Of these, nine cases (21%) harbored *NTRK1* fusions but the partner gene was unknown because of the method applied [17]. *NTRK1* fusions were found also in seven out of twenty-three (11%) Spitz nevi and in one out of four (25%) spitzoid melanomas [17]. Applying array comparative genomic hybridization (aCGH) as an ancillary analysis of diagnostically challenging melanocytic lesions, a search of the *NTRK*-fused cases was performed [14,15,16]. Yeh and colleagues identified three ASTs with the *ETV6-NTRK3* fusion and three ASTs with the *MYO5A-NTRK3* fusion (validated by DNA-based NGS, RNA-based NGS, and RT-PCR) in a series of 1202 difficult-to-classified melanocytic tumors [14]. Among the *NTRK3*-fused cases, there were two Spitz nevi [14]. The same author also reported 26 *NTRK1*-fused ASTs (confirmed using DNA-based NGS or RNA-based NGS) among which the fusion partners were *LMNA* in 11 cases and *TPM3, TP53*, and *KHDRBS1* each in one case [15]. *NTRK1* fusions were detected even in eight Spitz nevi and in four spitzoid melanomas [15]. De la Fouchardière and colleagues identified aCGH 22 ASTs with NTRK3 fusions; in particular, 13 cases harbored the *MYO5A-NTRK3* fusion, seven cases the *ETV6-NTRK3* fusion, and two cases the *MYH9-NTRK3* fusion [16]. This case series included three Spitz nevi and four melanomas with *NTRK3* fusions [16].

The aim of this study was to further assess the frequency of *NTRK* fusions in a large series of ASTs and the detection accuracy of a novel two-step approach. A comparison between RNA-based NGS, real-time RT-PCR, and FISH as confirmatory technique in the AST setting was performed. Spitz nevi were not investigated because of the lack of diagnostic and therapeutic relevance of *NTRK* fusions in such lesions. Spitz melanomas, instead, deserve a specific study once the appropriate analytical strategy to detect *NTRK* fusions has been established.

## 2. Results

### 2.1. Pan-Trk Immunohistochemistry

**Figure 1:**
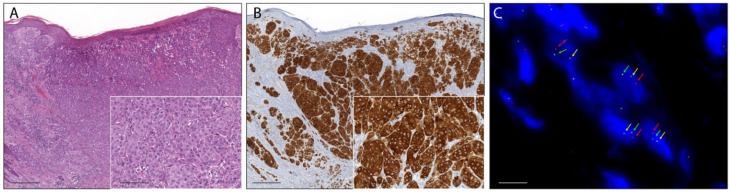
Photomicrographs of an atypical Spitz tumor harboring anNTRK1 fusion. (**A**): The lesion was an exophytic nodule with epidermal collarette, flat lower border, and focal epidermal hyperplasia with filigree-like rete ridges (H&E staining, magnification 40×, scale bar: 200 µm). Inset: lobulated nests of epithelioid melanocytes with distinct cell borders and quite pleomorphic nuclei (H&E staining, magnification 200×, scale bar: 40 µm). (**B**): Melanocytes showed strong and diffuse cytoplasmic immunoreaction (pan-TRK immunostaining, magnification 40×, scale bar: 200 µm). Inset: pan-TRK cytoplasmatic immunoreaction (pan-TRK immunostaining, magnification 200×, scale bar: 40 µm). (**C**): Break-apart FISH analysis confirmed NTRK1 rearrangement showing the typical pattern: one fusion signal (yellow arrows) and split signals 3′ (red arrows) and 5′ (green arrows) (ZytoLight SPEC NTRK1 Dual Color Break Apart Probe, original magnification 1000×, scale bar: 5 µm).

**Figure 2:**
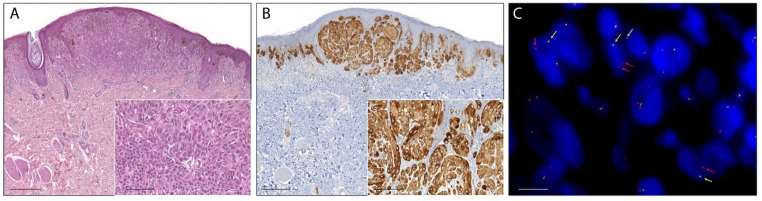
Photomicrographs of an atypical Spitz tumor harboring an NTRK1 fusion. (**A**): The lesion was dome-shaped with epidermal hyperplasia (H&E staining, magnification 40×, scale bar: 200 µm). Inset: lobulated nests of epithelioid melanocytes with distinct cell borders and quite pleomorphic nuclei showing a tendency towards maturation that became exaggerated at the bottom of the lesion (H&E staining, magnification 200×, scale bar: 40 µm). (**B**): Melanocytes showed strong and diffuse cytoplasmic immunoreaction (pan-TRK immunostaining, magnification 40×, scale bar 200 µm). Inset: pan-TRK cytoplasmatic immunoreaction (pan-TRK immunostaining, magnification 200×, scale bar 40 µm). (**C**): Break-apart FISH analysis confirmed NTRK1 rearrangement showing the atypical pattern: one fusion signal (yellow arrows) and a single orange signal 3′ (red arrows) (ZytoLight SPEC NTRK1 Dual Color Break Apart Probe, original magnification 1000×, scale bar 5 µm).

**Figure 3:**
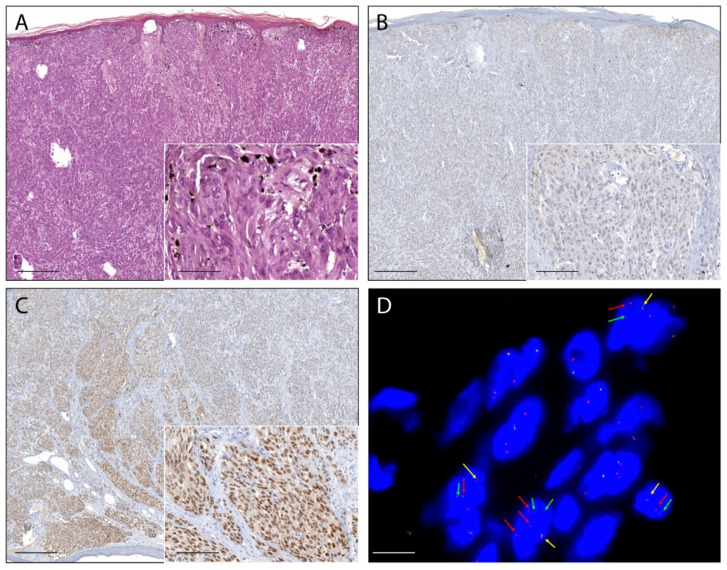
Photomicrographs of an atypical Spitz tumor harboring an NTRK3 fusion. (**A**): The lesion was an exophytic nodule with epidermal collarette, flat lower border, and widespread epidermal hyperplasia with long and thin filigree-like rete ridges enveloping superficial nests. (H&E staining, magnification 40×, scale bar 200 µm). Inset: melanocytes were predominantly elongated, with ground-glass cytoplasm, and arranged in fascicles (H&E staining, magnification 200×, scale bar 40 µm). (**B**): In some areas, the melanocytes lacked immunoreaction (pan-TRK immunostaining, magnification 40×, scale bar: 200 µm). Inset: melanocytes without immunoreaction (pan-TRK immunostaining, magnification 200×, scale bar 40 µm); (**C**): Most of the melanocytes showed strong nuclear immunoreaction (pan-TRK immunostaining, magnification 40×, scale bar: 200 µm). Inset: melanocytes with nuclear immunoreaction (pan-TRK immunostaining, original magnification 200×, scale bar: 40 µm). (**D**): Break-apart FISH analysis confirmed NTRK3 rearrangement showing the typical pattern: one fusion signal (yellow arrows) and split signals 3′ (red arrows) and 5′ (green arrows) (ZytoLight SPEC NTRK3 Dual Color Break Apart Probe, original magnification 1000×, scale bar 5 µm).

Fifteen percent (26/180) of ASTs showed a moderate to strong pan-Trk immunoreaction in melanocytes and were considered positive. In 24 cases (14%), the immunostaining was faint or barely perceptible and those cases were considered as negative. In additional five lesions (1%), the immunoreaction was not evaluated because of technical problems such as section detachment. In the remaining 125 ASTs (70%) pan-Trk immunoreaction in melanocytes was completely absent. Pan-Trk expression was observed in the cytoplasm in 24 cases (92%), with exclusive cytoplastic staining in eight cases (Figure 1 and Figure 2), in the nuclear membrane in 12 cases (46%), and in the nucleus in 11 cases (42%) with exclusive nuclear staining in two cases (Figure 3). No immunostaining was observed in the cellular membrane. Demographic, clinical and immunohistochemical data of the atypical Spitz tumors with positive pan-TRK immunoreaction are listed in Table 1. Histopathological details of the same cases are summarized in Table 2.

### 2.2. RNA-Based NGS

Total RNA was extracted from all the 26 ASTs with positive pan-TRK immunoreaction and from 31 ASTs with negative pan-TRK immunoreaction (23 with weak TRK expression and nine without immunostaining). In 22 cases (38% of samples; 11 cases from the positive group and 11 from the negative group) the quantity and quality of the RNA was not adequate for the molecular analyses. Libraries from the remaining 35 extracts were analyzed with the two RNA-based NGS methods to confirm the presence of NTRK1, NTRK2, and NTRK3 fusions. The two methods yielded concordant results. In 20 cases (10 cases from the positive group and 10 from the negative group) the libraries were not evaluable due to insufficient RNA amount. The LMNA-NTRK1 fusion was detected in four ASTs with positive pan-TRK immunoreaction while the remaining case showed the ETV6–NTRK3 fusion. No fusions were identified among the 10 cases with weak TRK expression and in cases without immunostaining.

### 2.3. Real-Time RT-PCR

Extracts analyzed with the RNA-based NGS technique were also tested with Real-Time RT-PCR. In 16 cases (six cases from the positive group and 10 from the negative group) the RNA was not evaluable. In three ASTs with positive pan-TRK immunoreactions there was a fusion involving the exon 10 of NTRK1, in another five ASTs a fusion involving the exons 11–12 of NTRK1, and in the remaining AST a fusion involving the exon 15 of NTRK3. All the fusions detected by RNA-based NGS were concordant with the results of RT-PCR. No fusions were detected in the 10 cases with weak or absent TRK expression. Molecular details of the 26 cases with positive pan-Trk immunoreaction are summarized in Table 3.

### 2.4. Fluorescence In Situ Hybridization

The break-apart FISH assay was used to further confirm the presence of NTRK1, NTRK2, or NTRK3 fusion in 23 ASTs with positive pan-TRK immunoreaction. In three cases the FFPE material was insufficient for further analysis. Overall, eight ASTs showed a signal pattern consistent with genomic alteration involving the NTRK1 locus. Three cases showed a typical break-apart pattern (Figure 1), and five cases showed an atypical pattern with one fusion signal and a single orange signal (Figure 2). The NTRK1 FISH test was not evaluable in five cases. No NTRK2 signal aberrations were detected in this series of ASTs. In one case the typical break-apart pattern indicative of NTRK3 rearrangement was observed (Figure 3).

## 3. Discussion

Recent advances in AST research have revealed a highly heterogeneous molecular landscape, very different from that of common nevi and malignant melanomas. ASTs usually harbor oncogenic mutations of *HRAS* or kinase gene fusions involving *ALK, BRAF, MET, NTRK1, NTRK3, RET,* and *ROS1* in a mutually exclusive pattern, whereas *BRAF* and *NRAS* mutations are absent [5,6,7,8,9,10,11,12,13,14,15,16,17]. This provided an opportunity for a molecular pathway-based tumor classification that can be very useful in the routine diagnostics [18]. Indeed, a worrisome melanocytic lesion with Spitz nevus features should lack *BRAF* and *NRAS* mutations and harbor one of these typical initiating genetic drivers to be classified as AST or Spitz melanoma [19]. In particular, the detection of appropriate molecular alterations allows identification of Spitz melanomas among the heterogeneous category of spitzoid melanomas, which includes lesions ranging from melanomas with tumor cells with spitzoid cytological features to melanomas showing epidermal hyperplasia and clefting around the melanocytic nests. It must be highlighted that many Spitz melanomas are diagnosed as such because of known synchronous metastasis or are initially diagnosed as ASTs and classified as fully malignant after the detection of distant metastasis during clinical follow-up. Indeed, it is well known that distinguishing AST from Spitz melanoma histologically is very difficult and at times impossible, even with the aid of common ancillary analyses. The promising impact of these discoveries to better understand tumor mechanisms and improve treatments is highlighted by the novel targeted drugs, larotrectinib and entrectinib, that could be used as therapy for *NTRK*-fused ASTs with symptomatic nodal involvement, as an alternative to surgery, or for *NTRK*-fused Spitz melanomas with distant metastasis detected during the follow up [20,21]. However, the prevalence of *NTRK* fusions among ASTs and the optimal detection approach has not been determined so far.

The present study identified *NTRK* fusions in the largest series of ASTs (*n* = 180) so far reported in the literature using different molecular techniques. The observed frequency of 9% is lower than that found by others but was based on a small sample size [13]. Of note, the fact that five pan-TRK positive cases in our study were not molecularly evaluable might have slightly reduced the prevalence of *NTRK* fusions in ASTs.

Regarding the type of fusions, in line with previous evidence most cases (15 out of 16; 94%) harbored *NTRK1* fusions and in one case an *NTRK3* fusion [13,14,15,16]. In four cases RNA-based NGS detected the *LMNA-NTRK1* fusions and in one case the *ETV6-NTRK3* fusion. In three cases RT-PCR highlighted a translocation of *NTRK1* involving the exon 10. However, the limit of this method is that it does not distinguish the specific fusion among those investigated (*SQSTM1-NTRK1, TFG-NTRK1, TPM3-NTRK1, TPR-NTRK1,* and *TRIM63-NTRK1)*. Likewise, for the translocation of *NTRK1* involving the exons 11–12, the precise fusion between *BCAN-NTRK1, LMNA-NTRK1, PPL-NTRK1,* and *TPR-NTRK1* remains undetermined. In another seven cases the *NTRK1* translocation was detected by FISH; thus, the fusion partner is unknown since this technique uses break apart probes flanking only *NTRK1* region. Regarding the available data, *LMNA-NTRK1* was the most common *NTRK1* fusion in this series of ASTs, according to the previous reports [13,15]. The chimeric protein product of this fusion is involved in the activation of the MAPK and PI3K pathways promoting proliferation and migration of the melanocytes [13,22,23]. The *ETV6-NTRK3* fusion, identified more than 20 years ago, is highly common in a series of different rare tumors and occasionally present in other more frequent neoplasms [24]. This translocation is known to be oncogenetic and constitutively signals always through the MAPK and PI3K pathways [14].

For tumors with uncommon *NTRK* fusions it is recommended to apply RNA-based NGS or, otherwise, to first screen the cases with immunohistochemistry and to confirm the positive cases with RNA-based NGS [25]. In the present study the reliability of the second strategy was assessed. Moreover, Real Time RT-PCR and FISH were evaluated as possible alternative confirmatory assays. For screening purpose, the expression of TRKA, TRKB, and TRKC must be detected simultaneously with a pan-TRK antibody, thus sparing material and time. The clone EPR17341 has been applied in melanocytic tumors showing a high sensitivity, a feature particularly useful in settings with a low prevalence of *NTRK* fusions such as ASTs [26,27,28,29]. Cases lacking pan-TRK immunoreaction are excluded from further molecular analyses. The present findings support this proposal, since pan-TRK immunohistochemistry was highly efficient in identifying *NTRK*-fused ASTs, as this clone demonstrated to be very specific in the previous and current study [29]. As for the subcellular localization of the immunoreaction and the different *NTRK* fusions, effectively *NTRK3* was detected in one case showing nuclear immunoreaction, as previously reported [16]. In brief, pan-TRK IHC is a reliable and effective first-line analysis to screen NTRK fusions in ASTs. Moreover, it is fast and relatively economic (it must be highlighted that the CE/IVD test also requires the use of the companion immunostainer).

RNA-based NGS is the gold standard analytic method according to current guidelines because it is extremely sensitive and specific [25]. It avoids the limitation of intronic coverage of DNA-based NGS since the introns are spliced out. The RNA-level directly confirms the functional transcription of the identified fusions. The sequence analysis permits determination of whether the chimeric protein would be translated and in-frame. Finally, some of the commercially available technologies also allow to characterize novel fusion partners of *NTRK1*, *NTRK2*, and *NTRK3* [30,31]. Another advantage is that tumors harboring *NTRK* fusions usually express at high levels the chimeric protein, allowing detection of translocation in the RNA of samples with low level of purity [30]. The main weakness of this technique is the lability of RNA, since RNA extracted from FFPE samples is frequently fragmentated and degraded through use of formalin and prolonged storage. This limit was also present in our retrospective study, as about 70% of cases showed poor quality, and the quantity of RNA being inadequate for the analyses. Other limitations of this time-consuming method are the costs, which are almost double the other two molecular methods, and its availability and diffusion in pathology laboratories.

RT-PCR can detect the presence of several *NTRK* fusions in a single run in the RNA extracted from a tumor sample (always depending on its quality and quantity) [30]. Although RT-PCR is more rapid and less expensive than NGS, *NTRK* fusion partners and breakpoints must be known *a priori* to be included in the panel of alterations identified by this technique as the primers must be specifically designed for each fusion recognized by the panel [30]. However, because in the ASTs settings the range of *NTRK* fusions is limited and already included in commercial panels, this does not seem to be a real issue.

FISH analyzes DNA to identify large structural variants such as oncogenic fusions and several break-apart probes for *NTRK1*, *NTRK2*, and *NTRK3* are already commercially available. The main strengths of FISH entail the direct view of the alterations in the cell nucleus, the small amount of required material (an unstained section for each probe used), the short (2-days) turnaround time, and the limited cost of the probes [30, 31]. The main disadvantage is the marked dependance from the expertise of the operator. If the interpretation of break-apart FISH assays is usually straightforward, occasionally it may be challenging, with the substantial risk of false negative results. Little inversions and intrachromosomal translocations caused by gene deletions may result in a short-split length of the signals flanking the gene of interest with break-apart probes (usually a distance greater than two signals is used as cut-off to confirm a translocation) or in the loss of a signal in the nuclei (known as atypical FISH pattern) [30,31,32]. This could be the case of *LMNA-NTRK1* fusion, the most common in ASTs, which is often formed through an intrachromosomal deletion involving *NTRK1* on chromosome 1 [13,33] An example of false negative result in an AST harboring such a fusion is reported in the present study. Nevertheless, FISH was the only method able to detect *NTRK1* fusions in the other seven cases, demonstrating its usefulness in archival FFPE samples. Interpretation of *NTRK* FISH relies on the minimum percentage of nuclei with signal alterations required to consider a tumor translocated. However, this value has not been fixed yet. Moreover, FISH does not allow identification of the fusion partner gene nor if the alteration of the *NTRK* gene results in a functional transcribed chimeric protein [30].

Considering the pros and cons of each technique, a two-step testing algorithm based on immunohistochemical screening (step one), followed by RNA-based NGS or RT-PCR confirmatory analysis (step two) in positive cases can be applied to ASTs. FISH should be performed in all molecularly unconfirmed cases. The main strengths of the present study lie in the collection and analysis of a large series of ASTs centrally reviewed and in the redundant use of solid and reliable molecular techniques. The main weaknesses of this study are the retrospective design and the involvement of multiple centers in the collection of the case series, two obligatory choices when dealing with a relatively rare disease such as AST. As mentioned above, nucleic acids of long stored FFPE archival materials are usually degraded, impacting on the results of molecular tests. However, in daily diagnostic practice, this may be less of a barrier when recent samples are analyzed. The highly different pre-analytical and conservation conditions of the FFPE samples across laboratories, while accounting for most of the technical difficulties, made results of this study closer to real-world situations.

In conclusion, the frequency of *NTRK* fusions in large series of ASTs was 9%, with a clear prevalence of *NTRK1* compared to *NTRK3* alterations. Pan-TRK immunohistochemistry is a fast, relatively cheap, and well-performing screening test that identifies ASTs that harbor *NTRK* fusions. Molecular confirmation of *NTRK* alteration may require the use of multiple techniques. Future studies using this two-step approach should determine the prevalence of *NTRK* fusions also in Spitz melanomas.

## 4. Materials and Methods

### 4.1. Case Selection and Tumour Specimen Collection

For this retrospective study, archival formalin-fixed and paraffin-embedded (FFPE) samples of 200 ASTs diagnosed during the period 1995–2019 were collected from ten Italian centers: University of Florence, Rome, University Hospital of Turin, University Hospital of Padua, Naples, Modena, Bergamo, Monza, Milan, Genoa, and Bologna. All cases were reviewed applying the diagnostic criteria stated by the fourth edition of the World Health Organization classification of skin tumors [32]. Upon review, 20 cases were reclassified as Spitz nevi or Spitz melanomas and thus excluded from the study.

### 4.2. Immunohistochemistry

Immunohistochemistry was performed on 4 μm paraffin-embedded whole tissue sections using standard techniques with the Ventana pan-TRK Assay (clone EPR17341; #790-7026, ready to use, Ventana Medical Systems, Tucson, AZ, USA), a rabbit recombinant monoclonal antibody reactive to a C-terminal epitope conserved across TRK-A, -B, and -C proteins and present in both wild-type and chimeric proteins. All assays were performed on a fully automated Ventana Discovery XT Immunostainer (Ventana Medical Systems, Tucson, AZ, USA). The staining protocol included pretreatment with cell conditioner followed by incubation with antibody. The signal for the antibody was then developed with Discovery anti-Rabbit HQ, Discovery Anti-HQ HRP, and CromoMap DAB (Ventana Medical Systems, Tucson, AZ, USA). After the staining run was complete, the tissue sections were counterstained with hematoxylin. Ganglia of the submucosal plexus of a normal vermiform appendix and an infantile fibrosarcoma with known *ETV6-NTRK3* fusion were used as positive controls and were run simultaneously. Keratinocytes, endothelial cells, and lymphocytes served as internal negative controls. Only moderate to strong immunostaining in melanocytes was considered positive. The subcellular localization of the labeling (i.e., nucleus, nuclear membrane, cytoplasm, and cellular membrane) was also evaluated.

### 4.3. RNA Extraction and Quantification

Hematoxylin and eosin-stained tumor slides were examined to identify representative areas of tumor suitable for molecular testing. RNA extraction from ASTs with positive pan-TRK immunoreaction, and in a subgroup of samples with weak or completely absent TRK expression was carried out according to the manufacturers’ protocols utilizing the MagCore Total RNA FFPE One-Step Kit (RBC Bioscience Corp., New Taipei City, Taiwan) on the automated extraction system MagCore Super (RBC Bioscience Corp. New Taipei City, Taiwan) based on magnetic beads extraction technology. RNA input quantification was measured by real-time PCR on an EasyPGX qPCR instrument 96 (Diatech Pharmacogenetics, Jesi, Italy), ensuring an accurate and precise measurement of the amplifiable RNA in the following amplification reaction of the target library regions. The assay allows the detection of two RNA regions highly conserved of 105 bp and 175 bp. The detection was performed using probes labelled with FAM and HEX, respectively. The RNA concentration was assessed by quantification with a standard curve in the HEX channel. The ratio between the quantification (ng/μL) obtained in FAM and that obtained in the HEX allows evaluation of the DNA fragmentation. Analysis of the assay was done by the dedicated EasyPGX Analysis Software (Diatech Pharmacogenetics, Jesi, Italy), that calculates the concentration and degree of fragmentation of the samples. Therefore, 10 tumor samples with an adequate quantity and quality of nucleic acids for sequencing were utilized for NGS library preparation.

### 4.4. RNA Libraries Preparation and NGS Sequencing

RNA libraries were generated using the Myriapod NGS Cancer panel RNA (Diatech Pharmacogenetics, Jesi, Italy), according to the manufacturers’ instructions. The kit allows the detection of the main gene fusions involving ten recurrently rearranged cancer genes: *ALK, ROS1, RET, NTRK1, NTRK2, NTRK3, FGFR2, FGFR3, PPARG* and the skipping of exon 14 of *MET* in total RNA isolated from tumor tissue. The RNA was retro-transcribed into cDNA using random hexamers. Subsequently, cDNA was amplified by multiplex-PCR using two primer mixtures to obtain fragments between 47 and 184 bases, including fusions of interest and endogenous control genes (PCR1). The amplification products were purified with magnetic beads to remove residual primers. An amplification-based indexing reaction (PCR2) followed, which allowed a unique pair of two sample-specific barcodes (indexes) and an Illumina platform-specific adapter to be attached to each fragment. The libraries thus constituted were normalized in quantity by magnetic beads to guarantee a homogeneous coverage of the samples during sequencing. Finally, the normalized libraries were mixed (library pool) and sequenced in parallel on the Illumina MiSeq platform (Illumina Inc., San Diego, CA, USA) with MiSeq Reagent Kit v2 Micro (300 cycles) flow cell (Illumina Inc., San Diego, CA, USA).

### 4.5. Sequencing Data Analysis

The data generated by the sequencer were analyzed locally with dedicated Myriapod NGS Data Analysis Software (v 4.0.2; Diatech Pharmacogenetics, Jesi, Italy).

### 4.6. Real-Time-PCR

The *NTRK1, NTRK2*, and *NTRK3* fusion analysis was also performed with the EasyPGX ready NTRK fusion kit (Diatech Pharmacogenetics, Jesi, Italy), an in vitro diagnostic test for the detection of fusions involving the three *NTRK* genes in the total RNA isolated from FFPE tumor sample and amplified by One Step RT-PCR. The detectable, but not distinguishable gene fusions, detected with this test are listed in Appendix A Table A1. The control group comprised a control assay. An expressed gene was amplified in the channel stably and independently from the original tumour tissue to verify the correct execution of the amplification process, the RNA quantity used and the possible presence of inhibitors that can cause false negative results. A positive and a negative control were also used. Detection took place via a fluorescent probe marked with FAM and HEX. It also included eight assays for the detection of gene alterations. Each of these provided simultaneous detection of the target through a probe marked with FAM, and an endogenous control gene through a probe marked with HEX. Data were analyzed by EasyPGX Analysis Software (Diatech Pharmacogenetics, Jesi, Italy).

### 4.7. Fluorescence In Situ Hybridization

To confirm gene fusion, the FISH assay was carried out on three 4 μm-thick sections cut from each AST FFPE sample with positive pan-TRK immunoreaction using the BOND FISH kit (Leica Biosystems, Newcastle Upon Tyne, UK) on the automated BOND system (Leica Biosystems) according to the manufacturer’s instructions. This kit consists of a formamide mixture to reduce nonspecific hybridization of nucleic acid probes. *NTRK1, NTRK2*, and *NTRK3* fusion detection was performed by three separate assays using specific break-apart probes for each gene: ZytoLight SPEC NTRK1 Dual Color Break Apart Probe (ZytoVision, Bremerhaven, Germany); ZytoLight SPEC NTRK2 Dual Color Break Apart Probe (ZytoVision, Bremerhaven, Germany); ZytoLight SPEC NTRK3 Dual Color Break Apart Probe (ZytoVision, Bremerhaven, Germany). Slides were counterstained with 4′,6-diamidine-2′-phenylindole dihydrochloride (DAPI) in antifade solution and examined using an automated CytoVision platform (Leica Biosystems, Newcastle Upon Tyne, UK). FISH interpretation was performed with the automated fluorescence microscope Leica DM5500 B (Leica Biosystems, Newcastle Upon Tyne, UK) using the filter ET-D/O/G for double Spectrum Green plus Spectrum Orange.

FISH signals were counted in at least 50 nonoverlapping intact nuclei. FISH was considered positive in relation to two different patterns: (a) a classic break-apart pattern with one fusion signal and two separated orange and green signals (separation distance of at least two signal diameters between the green and orange signals); (b) an atypical pattern with one fusion signal and a single orange signal without a corresponding green signal. No threshold regarding the percentage of nuclei showing one of these two signal patterns was applied to consider positive a case.

## Figures and Tables

**Table 1:** Demographic, clinical and immunohistochemical data of the atypical Spitz tumors with positive pan-TRK immunoreaction.

Case	Age (Years)	Sex	Re-Excision	Sentinel Lymph Node Evaluation	Events	Follow-up (Months)	Cytoplasm	Cellular Membrane	Nucleus
1	24	F	Yes	Negative	-	60	+	-	-
2	4	F	Yes	Not Evaluated	-	50	+	-	-
3	10	F	No	Not Evaluated	-	67	+	-	-
4	13	F	No	Not Evaluated	-	1	+	-	-
5	18	F	Yes	Positive	-	240	+	-	-
6	22	F	Yes	Not Evaluated	-	63	+	-	-
7	37	F	Yes	Negative	-	50	+	-	-
8	36	F	Yes	Negative	-	132	+	-	-
9	37	F	Yes	Negative	-	132	+	-	-
10	38	F	Yes	Not Evaluated	-	159	+	-	-
11	59	F	Yes	Not Evaluated	-	43	+	-	-
12	32	M	Yes	Not Evaluated	-	50	+	-	-
13	30	M	No	Not Evaluated	-	137	+	-	-
14	61	M	Yes	Negative	-	96	+	-	-
15	2	M	Yes	Not Evaluated	-	84	+	-	-
16	18	M	Yes	Not Evaluated	-	42	-	-	+
17	24	M	Yes	Not Evaluated	-	110	+	-	+
18	3	F	No	Not Evaluated	-	66	+	-	+
19	8	F	No	Not Evaluated	-	89	+	-	+
20	12	F	Yes	Not Evaluated	-	124	+	-	+
21	31	F	No	Not Evaluated	-	12	+	-	+
22	47	F	Yes	Negative	-	84	+	-	+
23	24	M	Yes	Not Evaluated	-	132	+	-	+
24	39	M	Yes	Negative	-	72	+	-	+
25	57	M	Yes	Negative	Local Recurrence	72	+	-	+
26	18	F	Yes	Negative	Local Recurrence	75	-	-	+

**Table 2:** Histopathology of the atypical Spitz tumors with positive pan-TRK immunoreaction.

Case	Site	Diameter (mm)	Thickness (mm)	Mitoses (Number/mm^2^)	Cytotype	Filigree-Like Rete Ridges	Lobulated Melanocytic Nests	Rosette-Like Structures
1	NA	24	1.5	0	Epithelioid	-	-	-
2	NA	4	1.9	4	Epithelioid	-	-	-
3	Leg	10	1.6	1	Epithelioid	+	-	-
4	Arm	13	0.7	1	Epithelioid	-	-	-
5	Ear	18	12	1	Epithelioid/Spindle	-	-	-
6	NA	22	0.5	0	Spindle	-	-	-
7	NA	37	0.9	2	Epithelioid/Spindle	-	+	-
8	Leg	36	0.8	1	Epithelioid	-	-	-
9	Leg	37	0.7	0	Epithelioid/Spindle	-	-	-
10	Thigh	38	1.1	0	Epithelioid/Spindle	-	-	+
11	NA	59	0.7	1	Epithelioid/Spindle	-	+	-
12	NA	32	1.1	1	Epithelioid/Spindle	-	+	-
13	Arm	30	1.2	1	Epithelioid/Spindle	+	-	-
14	Leg	61	1.2	2	Epithelioid/Spindle	-	-	-
15	Buttock	2	0.9	2	Epithelioid	-	-	-
16	Trunk	18	4	1	Epithelioid/Spindle	-	-	-
17	NA	24	1.9	2	Epithelioid/Spindle	-	-	-
18	Leg	3	1	3	Epithelioid/Spindle	+	+	-
19	Leg	8	1.1	1	Epithelioid	+	-	-
20	Leg	12	1.1	1	Epithelioid	+	-	-
21	Leg	31	0.8	1	Epithelioid/Spindle	-	-	-
22	Arm	47	1.2	2	Epithelioid	-	-	-
23	Arm	24	0.5	0	Epithelioid/Spindle	-	+	-
24	Hip	39	1.8	1	Epithelioid/Spindle	+	-	-
25	Trunk	57	0.8	0	Epithelioid/Spindle	-	-	-
26	NA	18	0.8	0	Spindle	-	-	-

NA = not available.

**Table 3:** Molecular features of the atypical Spitz tumors with positive pan-TRK immunoreaction.

Case	RNA-Based NGS	Real-Time RT-PCR	FISH	Fusion Partner
1	NA/I	NA/I	Positive	Unknown (NTRK1)
2	NE	Positive	Negative	Unknown (NTRK1 exon 10)
3	NE	NE	Positive	Unknown (NTRK1)
4	NA/I	NA/I	NA/I	-
5	NA/I	NA/I	NE	-
6	NE	Positive	Positive	Unknown (NTRK1 exon 10)
7	NA/I	NA/I	NA/I	-
8	NA/I	NA/I	NA/I	-
9	Positive	Positive	NE	LMNA-NTRK1
10	NA/I	NA/I	Positive	Unknown (NTRK1)
11	NA/I	NA/I	Positive	Unknown (NTRK1)
12	Positive	Positive	Positive	LMNA-NTRK1
13	NE	NE	Negative	-
14	NA/I	NA/I	Negative	-
15	Positive	Positive	Negative	LMNA-NTRK1
16	Positive	Positive	Positive	ETV6-NTRK3
17	NA/I	NA/I	Positive	Unknown (NTRK1)
18	NE	Positive	NE	Unknown (NTRK1 exon 10)
19	NE	NE	NE	-
20	NA/I	NA/I	Negative	-
21	NE	NE	Positive	Unknown (NTRK1)
22	NE	Positive	Positive	Unknown (NTRK1 exon 11–12 del)
23	NE	NE	Negative	-
24	NE	NE	Positive	Unknown (NTRK1)
25	Positive	Positive	Negative	LMNA-NTRK1
26	NA/I	NA/I	Negative	Unknown (NTRK1)

NA/I = not available/inadequate; NE = not evaluable.

## Data Availability

The data presented in this study are available on request from the corresponding author. The data are not publicly available due to privacy restrictions.

## Appendix A

**Table A1:** List of detectable but not distinguishable gene fusions with the Easy PGX ready NTRK fusion test.

*NTRK1* exon 9–10	*TFG-NTRK1* T5:N9
	*NFASC-NTRK1* N20:N10
	*NFASC-NTRK1* N21:N10
*NTRK1* exon 10	*SQSTM1-NTRK1* S4:N10
	*TFG-NTRK1* T5:N10
	*TPM3-NTRK1* T7:N10
	*TPR-NTRK1* T21:N10
	*TRIM63-NTRK1* T8:N10
*NTRK1* exon 11–12 del	*BCAN-NTRK1* B13:N11
	*LMNA-NTRK1* L11 del178:N11
	*LMNA-NTRK1* L2:N11
	*PPL-NTRK1* P22 del2181:N11
	*TPR-NTRK1* T6 del122:N12 del99
*NTRK1* exon 12	*MPRIP-NTRK1* M21:N12
	*MPRIP-NTRK1* M18:N12
	*MPRIP-NTRK1* M14:N12
	*SCYL3-NTRK1* S11:N12
	*TPM3-NTRK1* T7:N12
	*TPR-NTRK1* T6:N12
*NTRK2* exon 12–15	*AFAP1-NTRK2* A13:N12
	*VCL-NTRK2* V16:N12
	*TLE4-NTRK2* T7:N15
	*TRIM24-NTRK2* T12:N15
*NTRK2* exon 16–17	*AGBL4-NTRK2* A6:N16
	*QKI-NTRK2* Q6:N16
	*SQSTM1-NTRK2* S4:N16
	*STRN-NTRK2* S3:N16
	*SQSTM1-NTRK2* S4:N17
*NTRK3* exon 14	*ETV6-NTRK3* E4:N14
	*ETV6-NTRK3* E5:N14
*NTRK3* exon 15	*ETV6-NTRK3* E4:N15
	*ETV6-NTRK3* E5:N15

## References

[1] Massi D., De Giorgi V., Mandalà M. (2016). The complex management of atypical Spitz tumours. Pathology.

[2] Ferrara G., Cavicchini S., Corradin M.T. (2015). Hypopigmented atypical Spitzoid neoplasms (atypical Spitz nevi, atypical Spitz tumors, Spitzoid melanoma): A clinicopathological update. Derm. Pr. Concept.

[3] Gerami P., Busam K., Cochran A., Cook M.G., Duncan L.M., Elder D.E., Fullen D.R., Guitart J., LeBoit P.E., Mihm M.C. (2014). Histomorphologic assessment and interobserver diagnostic reproducibility of atypical spitzoid melanocytic neoplasms with long-term follow-up. Am. J. Surg. Pathol..

[4] Cerroni L., Barnhill R., Elder D., Gottlieb G., Heenan P., Kutzner H., LeBoit P.E., Mihm M., Rosai J., Kerl H. (2010). Melanocytic tumors of uncertain malignant potential: Results of a tutorial held at the XXIX Symposium of the International Society of Dermatopathology in Graz, October 2008. Am. J. Surg. Pathol..

[5] Gill M., Renwick N., Silvers D.N., Celebi J.T. (2004). Lack of BRAF mutations in Spitz nevi. J. Investig. Dermatol..

[6] Palmedo G., Hantschke M., Rütten A., Mentzel T., Hügel H., Flaig M.J., Yazdi A.S., Sander C.A., Kutzner H. (2004). The T1796A mutation of the BRAF gene is absent in Spitz nevi. J. Cutan. Pathol..

[7] Van Dijk M.C., Bernsen M.R., Ruiter D.J. (2005). Analysis of mutations in B-RAF, N-RAS, and H-RAS genes in the differential diagnosis of Spitz nevus and spitzoid melanoma. Am. J. Surg. Pathol..

[8] Pollock P.M., Harper U.L., Hansen K.S., Yudt L.M., Stark M., Robbins C.M., Moses T.Y., Hostetter G., Wagner U., Kakareka J. (2003). High frequency of BRAF mutations in nevi. Nat. Genet..

[9] Bauer J., Curtin J.A., Pinkel D., Bastian B.C. (2007). Congenital melanocytic nevi frequently harbor NRAS mutations but no BRAF mutations. J. Investig. Dermatol..

[10] Bastian B.C., LeBoit P.E., Pinkel D. (2000). Mutations and copy number increase of HRAS in Spitz nevi with distinctive histopathological features. Am. J. Pathol..

[11] Botton T., Yeh I., Nelson T., Vemula S.S., Sparatta A., Garrido M.C., Allegra M., Rocchi S., Bahadoran P., McCalmont T.H. (2013). Recurrent BRAF kinase fusions in melanocytic tumors offer an opportunity for targeted therapy. Pigment. Cell Melanoma Res..

[12] Yeh I., Botton T., Talevich E., Shain A.H., Sparatta A.J., de la Fouchardiere A., Mully T.W., North J.P., Garrido M.C., Gagnon A. (2015). Activating MET kinase rearrangements in melanoma and Spitz tumours. Nat. Commun..

[13] Wiesner T., He J., Yelensky R., Esteve-Puig R., Botton T., Yeh I., Lipson D., Otto G., Brennan K., Murali R. (2014). Kinase fusions are frequent in Spitz tumours and spitzoid melanomas. Nat. Commun..

[14] Yeh I., Tee M.K., Botton T., Shain A.H., Sparatta A.J., Gagnon A., Vemula S.S., Garrido M.C., Nakamaru K., Isoyama T. (2016). NTRK3 kinase fusions in Spitz tumours. J. Pathol..

[15] Yeh I., Busam K.J., McCalmont T.H., LeBoit P.E., Pissaloux D., Alberti L., de la Fouchardière A., Bastian B.C. (2019). Filigree-like Rete Ridges, Lobulated Nests, Rosette-like Structures, and Exaggerated Maturation Characterize Spitz Tumors With NTRK1 Fusion. Am. J. Surg. Pathol..

[16] De la Fouchardière A., Tee M.K., Peternel S., Valdebran M., Pissaloux D., Tirode F., Busam K.J., LeBoit P.E., McCalmont T.H., Bastian B.C. (2021). Fusion partners of NTRK3 affect subcellular localization of the fusion kinase and cytomorphology of melanocytes. Mod. Pathol..

[17] Amin S.M., Haugh A.M., Lee C.Y., Zhang B., Bubley J.A., Merkel E.A., Verzì A.E., Gerami P. (2017). A Comparison of Morphologic and Molecular Features of BRAF, ALK, and NTRK1 Fusion Spitzoid Neoplasms. Am. J. Surg. Pathol..

[18] Elder D.E., Massi D., Scolyer R.A., Willemze R. (2018). World Health Organization Classification of Skin Tumours.

[19] Raghavan S.S., Peternel S., Mully T.W., North J.P., Pincus L.B., LeBoit P.E., McCalmont T.H., Bastian B.C., Yeh I. (2020). Spitz melanoma is a distinct subset of spitzoid melanoma. Mod. Pathol..

[20] Drilon A., Laetsch T.W., Kummar S., DuBois S.G., Lassen U.N., Demetri G.D., Nathenson M., Doebele R.C., Farago A.F., Pappo A.S. (2018). Efficacy of Larotrectinib in TRK Fusion-Positive Cancers in Adults and Children. N. Engl. J. Med..

[21] Drilon A., Siena S., Ou S.I., Patel M., Ahn M.J., Lee J., Bauer T.M., Farago A.F., Wheler J.J., Liu S.V. (2017). Safety and Antitumor Activity of the Multitargeted Pan-TRK, ROS1, and ALK Inhibitor Entrectinib: Combined Results from Two Phase I Trials (ALKA-372-001 and STARTRK-1). Cancer Discov..

[22] Reuther G.W., Lambert Q.T., Caligiuri M.A., Der C.J. (2000). Identification and characterization of an activating TrkA deletion mutation in acute myeloid leukemia. Mol. Cell Biol..

[23] Truzzi F., Marconi A., Lotti R., Dallaglio K., French L.E., Hempstead B.L., Pincelli C. (2008). Neurotrophins and their receptors stimulate melanoma cell proliferation and migration. J. Investig. Dermatol..

[24] Cocco E., Scaltriti M., Drilon A. (2018). NTRK fusion-positive cancers and TRK inhibitor therapy. Nat. Rev. Clin. Oncol..

[25] Marchiò C., Scaltriti M., Ladanyi M., Iafrate A.J., Bibeau F., Dietel M., Hechtman J.F., Troiani T., López-Rios F., Douillard J.Y. (2019). ESMO recommendations on the standard methods to detect NTRK fusions in daily practice and clinical research. Ann. Oncol..

[26] Conde E., Hernandez S., Sanchez E., Regojo R.M., Camacho C., Alonso M., Martinez R., Lopez-Rios F. (2021). Pan-TRK Immunohistochemistry: An Example-Based Practical Approach to Efficiently Identify Patients With NTRK Fusion Cancer. Arch. Pathol. Lab. Med..

[27] Hechtman J.F., Benayed R., Hyman D.M., Drilon A., Zehir A., Frosina D., Arcila M.E., Dogan S., Klimstra D.S., Ladanyi M. (2017). Pan-Trk Immunohistochemistry Is an Efficient and Reliable Screen for the Detection of NTRK Fusions. Am. J. Surg. Pathol..

[28] Bourhis A., Redoulez G., Quintin-Roué I., Marcorelles P., Uguen A. (2020). Screening for NTRK-rearranged Tumors Using Immunohistochemistry: Comparison of 2 Different pan-TRK Clones in Melanoma Samples. Appl. Immunohistochem. Mol. Morphol..

[29] Uguen A. (2019). Spitz Tumors With NTRK1 Fusions: TRK-A and pan-TRK Immunohistochemistry as Ancillary Diagnostic Tools. Am. J. Surg. Pathol..

[30] Solomon J.P., Benayed R., Hechtman J.F., Ladanyi M. (2019). Identifying patients with NTRK fusion cancer. Ann. Oncol..

[31] Zito Marino F., Pagliuca F., Ronchi A., Cozzolino I., Montella M., Berretta M., Errico M.E., Donofrio V., Bianco R., Franco R. (2020). NTRK Fusions, from the Diagnostic Algorithm to Innovative Treatment in the Era of Precision Medicine. Int. J. Mol. Sci..

[32] Doebele R.C., Davis L.E., Vaishnavi A., Le A.T., Estrada-Bernal A., Keysar S., Jimeno A., Varella-Garcia M., Aisner D.L., Li Y. (2015). An Oncogenic NTRK Fusion in a Patient with Soft-Tissue Sarcoma with Response to the Tropomyosin-Related Kinase Inhibitor LOXO-101. Cancer Discov..

[33] Wiesner T., Kutzner H., Cerroni L., Mihm M.C., Busam K.J., Murali R. (2016). Genomic aberrations in spitzoid melanocytic tumours and their implications for diagnosis, prognosis and therapy. Pathology.

